# Effect of Monochromatic Red, Blue, and White Light on Reproductive Hormones of Male Donkeys During the Non-Breeding Season

**DOI:** 10.3390/ani16030490

**Published:** 2026-02-04

**Authors:** Muhammad Faheem Akhtar, Ayman Abdel-Aziz Swelum, Changfa Wang

**Affiliations:** 1 Liaocheng Research Institute of Donkey High-Efficiency Breeding and Ecological Feeding, College of Agriculture and Biology, Liaocheng University, Liaocheng 252059, China; 2 Department of Animal Production, College of Food and Agriculture Sciences, King Saud University, Riyadh 11451, Saudi Arabia; 3 Department of Theriogenology, Faculty of Veterinary Medicine, Zagazig University, Zagazig 44519, Egypt

**Keywords:** artificial LED light, donkey reproduction, plasma hormones

## Abstract

Red light suppresses melatonin and helps in improving reproductive efficiency in donkeys during the non-breeding season (November–February). In this study, the effects of red, blue, and white LED light were assessed. For this purpose, 40 adult Dezhou donkeys were divided into 4 groups, each receiving equal treatment for 40 days. All groups received 8 h of natural light. Additionally, the red group received 6 h of 50 lux of red LED light (468 nm) directed at a single eye. The blue group received 6 h of 50 lux of blue LED light (468 nm). The white group received 6 h of 50 lux of white LED light (468 nm), and the control group received only 8 h of natural sunlight. Blood samples were collected on the 21st, 28th, 34th, and 40th day of the experiment to analyze plasma hormone concentrations of progesterone (P4), Inhibin B (INH-B), Testosterone (T), Activin-A, Luteinizing Hormone (LH), Follicle Stimulating Hormone (FSH), Antimullerian Hormone (AMH), and Melatonin. In conclusion, red LED light directed at one eye showed the most promising results, elevating plasma hormone concentrations of testosterone (T), Activin A, LH, FSH, AMH, and melatonin.

## Simple Summary

The donkey industry is one of the most vibrant animal farming industries in China. A lot of research is being conducted to improve various aspects, including reproduction. As a seasonal breeder, seasonality in breeding is a hurdle to achieving optimized production, including higher pregnancy rates and foal production. In recent years, researchers adopted various techniques to improve conception rates in Jennies, including estrus synchronization and inhibin immunization. The current study aimed to investigate the impact of various monochromatic light sources, i.e., red, blue, and white light on plasma hormones of Dezhou jennies during the non-breeding season (November–February). Our results showed that red light improves reproductive efficiency in jennies.

## 1. Introduction

In non-equatorial regions of Earth, seasonal breeders time reproduction to coincide with favorable seasons. Seasonal breeding is an adaptive quality adopted by animals in response to different geographical conditions. The photoperiod influences reproduction in ruminants at all latitudes [1]. All domesticated farm animals are derived from wild ancestors. In temperate zones, mammalian reproductive activity synchronizes with the breeding season. This synchronization enhances progeny survival and matches food availability [2]. Like the stallion [3], the hypothalamus–pituitary–gonadal axis in donkeys is influenced by photoperiod. Seasonality in reproduction is an unwanted complication in sustainable animal farming and must be circumvented.

In China, equine exhibit marked seasonal reproductive activity driven primarily by changes in photoperiod, with more pronounced effects at temperate and northern latitudes [4]. Male donkeys show clear seasonality in reproductive function, characterized by increased testicular size, testosterone secretion, libido, and semen quality during the long-day months of spring and summer [5]. Conversely, during the short-day months of autumn and winter, male donkeys experience reduced testicular activity, lower circulating testosterone concentrations, decreased sperm output, and compromised semen quality. This seasonal pattern is more evident in northern regions of China, where day length variation is greater [4,6].

Melatonin plays a major role in controlling biological rhythms in animals [7]. Circadian rhythms control physiological activities [8]. Melatonin is considered the gold standard for modulating circadian rhythm [9]. It controls reproduction by regulating the expression of circadian rhythm genes [10]. Thus, controlling melatonin secretion may modulate seasonal breeding. Eco-friendly photoperiodic treatments have been used in small ruminants, camels, and poultry (such as geese and layers) to prevent seasonal reproductive disorders [11,12]. Artificial light exposure advanced reproductive activity in mares, decreasing melatonin secretion. Photoperiod treatments can shorten the anestrus season in ewes and goats [13,14]. Scientists and research professionals working in assisted reproductive technologies put great effort into ensuring minimum sperm damage and maximizing fertilizing capacity while processing semen [15]. Red-light stimulation elevated mitochondrial and motility activity of fresh and post-thaw semen quality in donkeys without affecting their viability [16]. Similarly, mitochondrial function was modulated through the activity of the electron chain in donkey sperm after red light stimulation [17].

Mares exposed to 16 h of light and 8 h of dark ovulated earlier [18]. Donkeys are considered neglected animals [19]. More research has been conducted on horses than on donkeys in previous decades. Light programs in pregnant mares affect fetal maturation and growth [20]. Blue light is particularly effective in suppressing melatonin secretion in horses and advancing the ovulatory season in mares [21,22]. A blue light mask during the last trimester reduced gestation length in mares [23]. Polychromatic blue and red light at night increased testosterone levels in stallions [23]. Research has examined the effects of monochromatic blue light on pregnant mares [18] and on the resumption of estrus and ovulation in thoroughbred mares [24]. A large body of literature addresses the effects of artificial photoperiods and LED blue light in mares, but none exists on plasma hormones or the reproductive efficiency of various colored lights on male reproduction.

This study aimed to evaluate the effect of red, blue, and white LED light on plasma hormone concentrations (Activin A, AMH, E2, FSH, Inhibin-B, LH, Melatonin, P4, and T) in male donkeys during the non-breeding season.

## 2. Materials and Methods

### 2.1. Experimental Design

The experiment was conducted during the short-day, non-breeding season in China and lasted for 40 days. A total of 40 healthy adult male Dezhou donkeys were used. Before the start of the trial, all animals were acclimatized to Equilume head-mounted light masks, and no health-related or behavioral abnormalities were observed during the acclimation or experimental period.

All donkeys were maintained under identical environmental conditions, feeding regimes, and husbandry practices throughout the study. Animals were offered silage and had free access to drinking water. The experiment was carried out at Liaocheng Wanshixing Breeding Co., Ltd. (115° E, 36° N), Liaocheng, Shandong Province, China. The non-breeding season in this region is typically associated with reduced reproductive hormone activity and lower semen quality in males.

**Figure 1:**
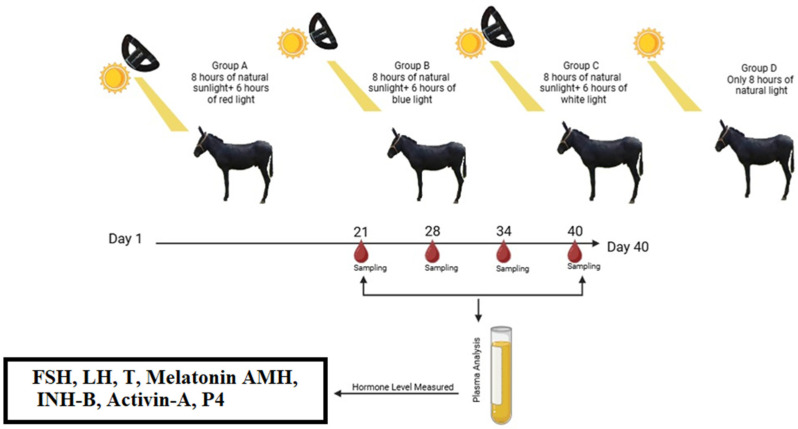
Illustration of experimental design.

After acclimatization, the 40 donkeys were randomly assigned to four groups (*n* = 10 per group). All groups received 8 h of natural daylight daily. In addition, three treatment groups were exposed to extended photoperiods using LED light delivered to a single eye for an additional 6 h at 50 lux. The red-light group received red LED light, the blue-light group received blue LED light, and the white-light group received white LED light. The control group received only natural daylight without photoperiod extension (Figure 1). Because environmental and management conditions were identical across all groups, the animals were submitted to the same environment [4].

### 2.2. Plasma Hormonal Analysis

Blood samples were collected for plasma hormone analysis and antibody titre. Blood samples were collected on the 21st, 38th, 34th, and 40th day of the light treatment experiment via the jugular vein into heparinized tubes. Within 3 h of sample collection, plasma was separated from blood by centrifugation at 1000× *g* and stored at −20 °C until analysis. Plasma progesterone (P4), Inhibin B (INH-B), Testosterone (T), Activin A, Luteinizing Hormone (LH), Follicle Stimulating Hormone (FSH), Antimullerian Hormone (AMH), and Melatonin concentrations were measured by ELISA (enzyme-linked immunosorbent assay) using quantitative kits (MEIMIAN, Jiangsu Meimian Industrial Co., Ltd., Nanjing, Jiangsu, China) [4]. Assays were performed according to the protocols provided by the kit supplier. For FSH (follicle-stimulating hormone), assay sensitivity was 0.075 U/L. The inter- and intra-assay coefficients of variation were below 10%. The detection range was 0.3–18 U/L. For plasma LH (luteinizing hormone), assay sensitivity was 0.005 ng/mL, and coefficients of variation were below 10%. The detection range was 0.002 ng/mL to 0.05 ng/mL. For progesterone (P4), assay sensitivity was 5 pmol/L. Inter- and intra-assay coefficients were below 10%. The detection range was 20–800 pmol/L. For testosterone (T), assay sensitivity was 0.02 ng/mL. Inter- and intra-assay coefficients were below 10%. The detection range was 0.094 ng/mL to 3.77 ng/mL. For AMH (anti-Müllerian hormone), assay sensitivity was 0.05 ng/mL. Inter- and intra-assay coefficients were below 10%. The detection range was 0.2–8.5 ng/mL. For Activin (A), assay sensitivity was 0.4 ng/mL. Inter- and intra-assay coefficients were below 10%. The detection range was 1.6–65 ng/mL. Assay sensitivity for estradiol (E2) was 1 pmol/L, and the detection range was 4–120 pmol/L. Inter- and intra-assay coefficients were below 10%. The assay sensitivity for INH-B (inhibin B) was 2.5 pg/mL. The detection range was 10–700 pg/mL, and intra-assay coefficients were below 10%. Assay sensitivity for Melatonin was 0.3 ng/L. The detection range was 1.2–45 ng/L, and intra-assay coefficients of variation were below 10%.

### 2.3. Statistical Analysis

All data was analyzed with R software (version 4.3.2; R Foundation for Statistical Computing, Vienna, Austria). Normality and homogeneity were tested using the Shapiro–Wilk and Levene Tests, respectively.

Each response variable was then fitted separately using a linear mixed model (LMM, a statistical model with both fixed and random effects) in the lme4 package(version 1.1-37). Treatment (A, B, C, and D), week, and their interaction were included as fixed effects. Treatment was a random effect. The model was represented as:

Yijk = m + Ti + Wj + (T × W)ij + Ak + eijk

With respect to the concentration of the hormone (Yijk), the general mean is (m), the treatment effect is (Ti), the week effect is (Wj), the animal random effect is (Ak), and the error is (eijk).

The emmeans package (version 1.11.2-8)was used to run post hoc pairwise tests with the HSD test at *p* = 0.05. The correlations between hormones were performed using Hmisc (Hormonal Pearson correlations) and correlation matrices (corrplot package). PCA multivariate relationships integrated hormonal and metabolic variables across treatments, using the Facto Mine R package(version 2.0.1). Ggplot2 drew charts with the same scale to facilitate comparison of variables.

## 3. Results

### 3.1. Overview of Photo-Stimulation Outcomes

**Figure 2:**
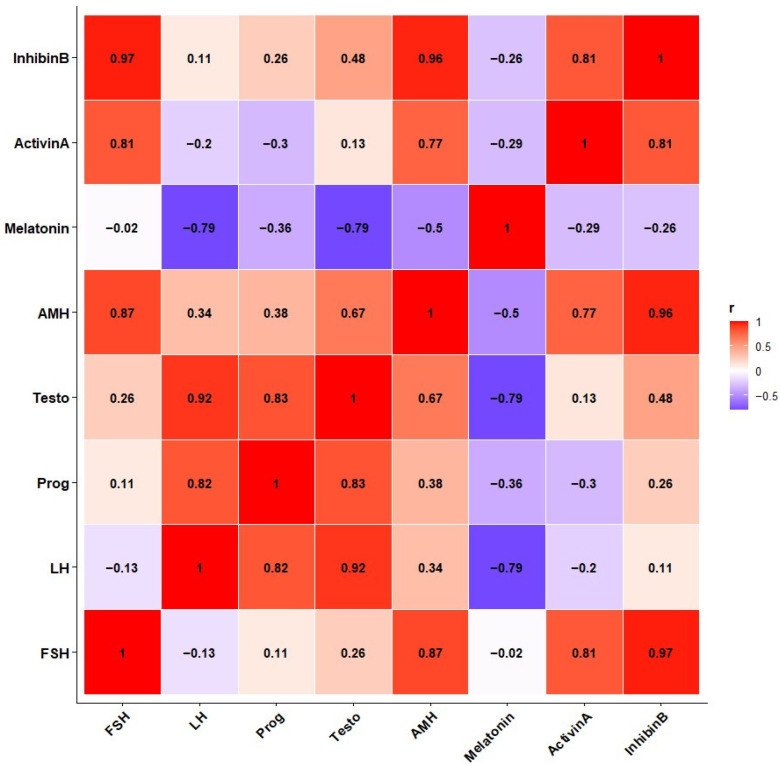
Correlation of the level of reproductive hormones. Heatmap of Pearson correlation coefficients of all measured hormones (FSH, LH, Progesterone, Testosterone, Melatonin, AMH, Activin A, and Inhibin B). Correlations between variables are represented by red (positive) and blue (negative), and the intensity shows the degree of association. The matrix highlights inter-hormonal relationships that depend on experimental conditions.

During the 40-day photoperiodic trial, no observable health or behavioral issues were noted. Baseline melatonin profiles showed the study started during a short-day non-breeding season. Artificial spectrum lighting successfully simulated a long-day signal. This resulted in sustained hormone secretion throughout the trial, as detailed in Figure 2.

### 3.2. Suppression of Nocturnal Melatonin by Spectral Light Exposure

Exposure to evening illumination suppressed blood melatonin levels in animals (Table 1). The control group had high morning concentrations (82.4 ± 4.5 pg/mL), reflecting endogenous night secretion. In contrast, exposure to blue, red, or white light lowered levels by 33%, 27%, and 29%, respectively (*p* < 0.01). The drop occurred after 1 week (melatonin × week, *p* < 0.05) and remained stable thereafter.

According to the correlation network (Figure 2), melatonin functioned as the central negative node. It showed an inverse relationship with pituitary and gonadal hormones. Baseline melatonin profiles confirmed that the study commenced during a short-day, low-reproductive phase. Artificial spectrum lighting was used to simulate a long-day signal during the short-day season. This intervention resulted in sustained hormone secretion over the experimental period, as shown in Figure 2. The degree of suppression matched findings in mares exposed to blue LED masks [18] and wild wallabies exposed to artificial light at night [25]. These results indicate that localized ocular illumination can influence the pineal gland’s activities in donkeys.

### 3.3. Pituitary Activation Following Melatonin Reduction

**Figure 3:**
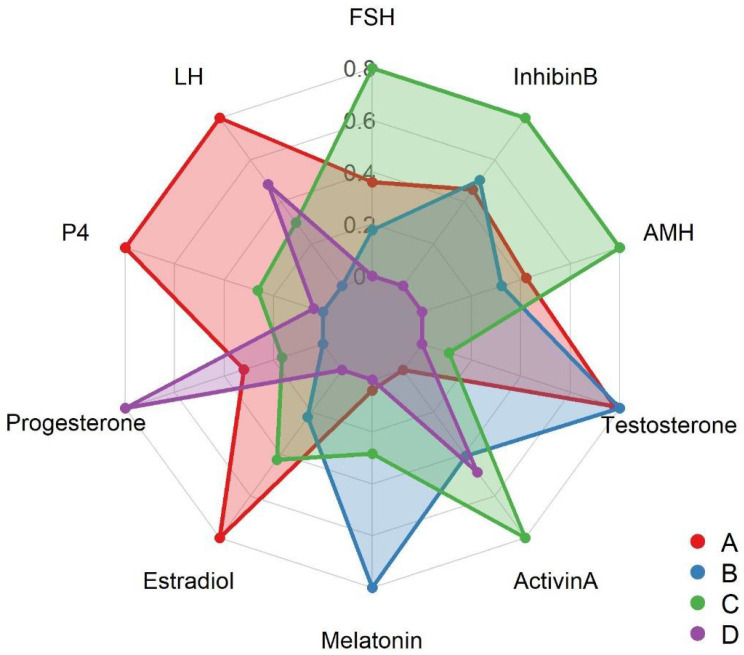
Radar plot of normalized hormone profiles of treatment groups. Radar chart of normalized levels of the hormones in the Z-score by red, blue, white, and control groups. The polygons denote each treatment group, and the multivariate patterns and differences in hormone levels caused by different light-exposure regimes.

The increase in pituitary gonadotropins (Table 1) resulted from chronic melatonin suppression. Average FSH increased from 1.12 ± 0.08 mIU/mL in controls to 1.65 ± 0.10 (red light, *p* = 0.032) and 1.36 ± 0.09 (white light, *p* < 0.05). Blue light increased FSH to 1.29 ± 0.07, which is lower than the above numbers. LH also increased from 2.45 ± 0.15 to 3.20 ± 0.22 (red) and 2.88 ± 0.19 (white) mIU/mL (*p* = 0.045). Radar charts (Figure 3) show that red-light animals had the highest increase in hormonal pituitary vertices.

Correlation analysis, as shown in Figure 2, demonstrated significant inverse relationships between melatonin and LH (r = −0.79, *p* < 0.01), whereas the correlation with FSH was weak and non-significant (r = −0.02, *p* > 0.05). These findings agree with the known fact that, unlike stallions, melatonin increases blood melatonin concentration in rams [22,26,27].

### 3.4. Stimulation of Gonadal Endocrine Activity

Pituitary gland stimulation and photo-stimulation were performed downstream of the pituitary, and, significantly, photo-stimulation improved testicular endocrine function. The testosterone concentration was increased by 54% under red light (5.40 ± 0.42 ng/mL vs. 3.50 ± 0.30 in controls; *p* = 0.021) and slightly under white light (4.20 ± 0.38 ng/mL. The red-light exposure also led to the elevation of Sertoli and Leydig cell peptides: Inhibin B (90.8 ± 5.6 vs. 72.1 ± 5.0 pg/mL; *p* = 0.036) and Activin A (74.6 ± 4.9 vs. 62.5 ± 4.3 pg/mL; *p* = 0.049).

**Figure 4:**
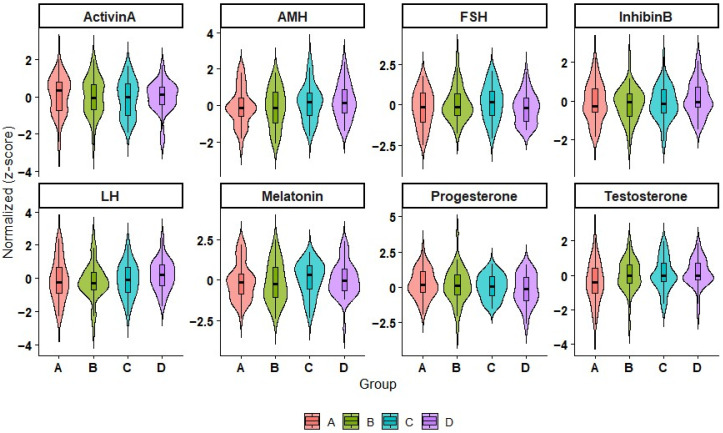
Normalized hormone levels distribution between treatment groups. Violin plots depicting the distribution and density of the normalized (Z-value) concentrations of the eight hormones in Groups A, B, C, and D. The embedded boxplots used to depict the median and interquartile ranges and reflect variability and effects of the treatment on the endocrine parameters.

AMH was increased significantly solely under white light (1.15 ± 0.05 ng/mL; *p* = 0.041), which reflects the boosted Sertoli-cell function that occurred in the late phase of activation. The violin plots in the reflections of Figure 4 show redistribution of testosterone, LH, and FSH, underscoring the uniform endocrine responses under red light. The combination of these findings suggests a joint resumption of function of the hypothalamic–pituitary–gonadal (HPG) axis after melatonin depression light, as observed in other long-day breeders, with seasonal reactivation patterns [28].

### 3.5. Metabolic Adaptation to Photo-Stimulation

Oxidative metabolism was also affected by exposure to spectral light. Plasma coenzyme) concentrations were increased significantly from 0.44 ± 0.02 µmol/L in controls to 0.60 ± 0.03 (red), 0.53 ± 0.03 (blue), and 0.50 ± 0.02 (white) (*p* = 0.025). correlated with testosterone (r = 0.61, *p* < 0.05) and Activin A (r = 0.55, *p* < 0.05) [29,30].

### 3.6. Integration of Endocrine and Metabolic Signals

The correlation heatmap revealed a compact clustering of pituitary, gonadal, and metabolic markers after photostimulation, as shown in Figure 2. The FSH, LH, testosterone, Activin A, and Inhibin B formed a strongly positive cluster (r = 0.81–0.97, *p* < 0.01), whereas melatonin was negatively loaded. The AMH was also tightly associated with Inhibin B (r = 0.96), confirming the strong Sertoli-cell coactivation.

The linkages detailed above were the ones agreed to by the Principal Component Analysis (Table 2): PC1 (47.9%) defined the axis of reproductive activation (high LH/FSH/testosterone/Activin A/Inhibin B vs. low melatonin); PC2 (23.7%) showed that Sertoli-cell function (AMH/Inhibin B) was inversely connected PC3 (12.8%). Animals exposed to red light achieved higher PC1 scores, blue-light animals showed similarity with PC3, and controls were grouped at low PC1 values, thereby confirming the specific wavelength-induced hormonal reorganization by light exposure.

### 3.7. Quantitative Linkage Between Melatonin and Reproductive Output

Regression analyses show that melatonin is a significant negative predictor of both gonadotropin and steroid levels. A decrease of 1 pg/mL in melatonin would lead to an increase of 0.32 mIU/mL in LH (β = −0.031 ± 0.006, *p* < 0.001) and a 10 pg/mL increase in testosterone (β = −0.046 ± 0.009, *p* < 0.001; R^2^ = 0.57–0.63).

### 3.8. Comparative Efficacy of Light Spectra

Among all treatments, blue light (≈468 nm) caused the most significant suppression of melatonin but had relatively minor gains in gonadotropic function, suggesting limited hypothalamic relay activation. Red light (625–650 nm) caused the most outstanding reproductive responses, with the highest levels of LH, FSH, testosterone, and Inhibin B. The effects of white light (400–700 nm) were in the middle. In this way, blue wavelengths are the most effective at inhibiting pineal secretion, whereas red light is the most effective at synchronizing the HPG axis and its related metabolism. The distribution of effects across wavelengths is as expected, consistent with equine and ovine photostimulation studies [22,26,27].

### 3.9. Network Reorganization and Seasonal Reactivation

Correlation-structure analysis demonstrated that photostimulation induced hormonal networks from a quiescent, melatonin-dominated configuration to an active, gonadotropin-centered one. During short days, melatonin overrode pituitary and gonadal function through its strong inhibitory effects. By light treatments, these negative couplings weakened and then changed into strong positive correlations between LH, FSH, testosterone, and Inhibin B (r = 0.81–0.97, *p* < 0.01). Such a reorganization is akin to the network transitions observed during natural spring reactivation in seasonal breeders, which provide evidence for the successful artificial replication of the long-day endocrine state.

### 3.10. Quantitative Summary

Of all the hormones, red-light exposure caused the greatest proportional changes compared to the controls: melatonin −27%, LH +31%, FSH +47%, testosterone +54%, Inhibin B +26%, Activin A +19%, +36%. Blue light was responsible for even greater melatonin inhibition (−33%), but lower increases in gonadotropins, thus confirming the partial decoupling of the pineal and pituitary responses. The results together show that red light is the most potent agent in fully transforming the endocrine system from a winter-hibernating, shut-off state to a summer-like activated state.

### 3.11. Principle Findings

With the help of Equilume masks, evening light simulated artificial light under which animals experienced significant suppression of melatonin secretion (Table 1, Figure 2), accompanied by proportional increases in gonadotropins and testosterone (Table 1, Figure 2). The Sertoli-cell biomarkers (AMH, Inhibin B increased at the same time as the reproductive activation began (Table 1). PCA (Table 2) indicated that the melatonin–gonadotropin axis primarily accounted for the variability in the data, while red-light treatment elicited the most potent endocrine activation. The work thus demonstrates that photostimulation at specific wavelengths can manipulate the HPG axis in inactive male donkeys during the season.

### 3.12. Consistency Summary of Endocrine Responses

The integrated evaluation of photostimulation outcomes, correlation structures, and PCA confirmed the coherence of the observed endocrine responses in male Dezhou donkeys. The suppression of melatonin by evening spectral light was consistently associated with proportional increases in pituitary and gonadal hormones. The correlation heatmap revealed that melatonin showed a strong negative association only with LH (r = −0.79, *p* < 0.01), whereas its association with FSH was weak (r = −0.02), suggesting that the hypothalamic relay primarily mediates gonadotropic activation via LH. The gonadotropins (LH and FSH), testosterone, Activin A, and Inhibin B formed a unified positive cluster (r = 0.81–0.97, *p* < 0.01), illustrating the synchronized activation of the hypothalamic–pituitary–gonadal (HPG) axis. Additionally, the Sertoli-cell markers AMH and Inhibin B showed the highest interdependence (r = 0.96), indicating the parallel reactivation of spermatogenic support function during light exposure. Upregulation of AMH and Inhibin B after red, blue, and white light stimulation indicates an increase in testicular size.

Principal component analysis further substantiated these associations. PC1, representing reproductive activation, integrated LH, FSH, testosterone, Activin A, and Inhibin B as dominant positive loadings against melatonin, accounting for 47.9% of the total variance. PC2, which explained 23.7% of the variance, reflected the coupling between Sertoli-cell activity (AMH/Inhibin B) and metabolic adaptation. Together, these results demonstrate that the hormonal and metabolic responses were internally consistent across all analytical levels, confirming that red light—by inducing the highest PC1 scores and the strongest positive network clustering—most effectively transitioned the animals from a suppressed, melatonin-dominant winter state to an activated, long-day endocrine phenotype.

## 4. Discussion

This research demonstrates the feasibility of regulating seasonal reproduction in donkeys through targeted light manipulation that combines photoperiodic entrainment and photobiomodulation. These findings are the first step towards the design of improved lighting systems, the management of energy, and the further integration of night biology with livestock production for the benefit of producers and the environment. The current research provides the initial scientific evidence that wavelength-specific *Equilum^®^* light masks can influence the pineal–hypothalamic–pituitary–gonadal (HPG) axis in male Dezhou donkeys during the non-breeding season. During two months of the controlled photo-stimulation trial, plasma melatonin was considerably decreased (Table 1; Figure 2), while pituitary (LH, FSH) and gonadal (testosterone, Inhibin B, Activin A) hormones increased correspondingly (Table 1; Figure 2 and Figure 3).

The demonstrated endocrine reorganization (Table 2) reflects physiological changes that naturally drive spring activation in long-day breeders such as horses [31]. Therefore, the study shows that providing artificial light from a single eye allows male donkeys to restart their reproductive axis without changes in management or diet.

The most crucial outcome of spectral photo-stimulation was a substantial decrease in nocturnal melatonin, confirming that ocular exposure to short- and long-wavelength light overrides the intrinsic biological clock (Figure 2). Blue light (≈468 nm) induced the highest suppression (–33%), matching the known peak sensitivity of melanopsin-expressing retinal ganglion cells [21,25]. These cells project to the suprachiasmatic nucleus, which regulates pineal melatonin rhythm [32]. The reduction in melatonin amplitude and duration mimics a “long-day” signal, thereby shifting the reproductive system from a winter-dormant to a summer-active state [22].

The reduction in melatonin levels detected after only one week demonstrates the rapid entrainment achievable with monochromatic light, comparable to the 7–10 days required in mares under similar *Equilume* protocols [18]. The magnitude of suppression (27–33%) is consistent with earlier findings in horses and sheep [26,27], indicating that pineal sensitivity is evolutionarily conserved among long-day–adapted ungulates.

Following melatonin suppression, LH and FSH displayed marked increases (Table 1; Figure 2). These elevations reflect upregulation of hypothalamic GnRH pulse frequency, as melatonin typically inhibits GnRH neuronal activity through MT_1_/MT_2_ receptor–mediated pathways (24, 25). The proportional increases in LH (+31%) and FSH (+47%) align with the early photo-stimulatory phase observed in rams and stallions, where GnRH activation precedes testicular response by 1–2 weeks.

The correlation structure further confirmed these interactions. Figure 2 shows that melatonin had a strong negative association only with LH (r = −0.79, *p* < 0.01), whereas the correlation with FSH was weak and non-significant (r = −0.02, *p* > 0.05). Conversely, LH, FSH, testosterone, Activin A, and Inhibin B formed a tight positive cluster (r = 0.81–0.97, *p* < 0.01), confirming the synchronized reactivation of the HPG axis. The observed interindividual uniformity in red-light animals signifies coherent neuroendocrine synchronization, a key requirement for herd-level reproductive alignment.

Red light produced the greatest elevation in testosterone (+54%), indicating that Leydig cell steroidogenesis was strongly stimulated (Table 1; Figure 2). This observation aligns with increased LH concentrations driving the activation of cAMP–P450scc and 17β-HSD enzymes, which are essential for androgen biosynthesis [7]. Inhibin B and Activin A rose concurrently, signifying Sertoli-cell reactivation and reinstatement of testicular paracrine signaling [27]. Our results are consistent with a previous study that found that sexual maturity promoted monochromatic red-light stimulation.

Monochromatic red light stimulation may have stimulated deeper photoreceptors [33]. The connection between increased gonadal hormone output and enhanced mitochondrial activity indicates that spectral light, especially red light, can elevate mitochondrial capacity and antioxidant levels, as shown in studies on photo-biomodulation in livestock spermatozoa [29,30]. Our results are in accordance with a previous study in which red light improved sperm motility and mitochondrial activity in donkeys [16]. Similarly, red light stimulated sperm’s mitochondrial function by affecting electron transport chain activity in donkey sperm [17]. Although we did not analyze semen quality in our study, mounting evidence suggests that an elevation in plasma hormone concentrations of T, LH, and FSH can improve semen quality parameters in horses and donkeys [34].

AMH increased moderately, especially under white light (Table 1), suggesting ongoing Sertoli maturation toward active spermatogenesis. These concurrent changes validate the classical hormonal cascade known in seasonal breeders (18, 24).

The red-light group (Figure 4) showed uniform hormone responses, likely due to longer wavelengths penetrating deeper into tissues, facilitating testicular stimulation via photo-biomodulation of mitochondrial cytochrome *c* oxidase [29,35].

Melatonin serves as an antioxidant [32]. Thus, melatonin reduction may transiently decrease pineal antioxidant capacity. The absence of a direct melatonin correlation (*p* = 0.18; Table 2) supports the notion of an independent peripheral photo-biomodulation mechanism complementing central neuroendocrine effects.

Spectral differences highlight distinct physiological pathways. Blue light, though most effective at melatonin suppression, elicited lower gonadotropic and steroidal responses, implying that pineal inhibition alone is insufficient for full gonadal activation. Conversely, red light induced the most pronounced hormonal and metabolic changes, signifying dual activation through both neuroendocrine disinhibition and tissue-level photo stimulation (Table 1; Figure 3 and Figure 4). White light elicited intermediate effects, possibly reflecting the combined influence of both inhibitory (blue) and stimulatory (red) wavelengths within its broad spectrum.

Principal component analysis (Table 2) and correlation mapping (Figure 2) illustrate the hierarchical reorganization of endocrine interactions following photo stimulation. PC1 (47.9% variance) represented the melatonin–gonadotropin–testosterone axis, while PC2 and PC3 described Sertoli–metabolic coupling and oxidative–circadian interplay, respectively. These findings align with known multivariate endocrine adjustments in long-day breeders.

The clustering of gonadotropins and testosterone underscores their integrated regulatory network, where mitochondrial metabolism underpins steroidogenic output. Animals exposed to red light achieved the highest PC1 loadings, corresponding to the most activated reproductive state, while controls remained at low PC1 values, indicative of winter quiescence. This organization confirms that photo stimulation not only alters absolute hormone levels but also reconfigures the architecture of the HPG axis.

The responses in Dezhou jack donkeys resemble those documented in horses exposed to long-day photoperiods or melatonin antagonism [22,26,27]. In rams and goats, melatonin increases reproductive activity. The slightly lower response magnitude compared to stallions may relate to species-specific photoperiodic sensitivity and the latitude of adaptation [28].

Red-light–treated males exhibited a more uniform hormonal distribution (Figure 3) than that reported for mares [18], suggesting that males may have a lower activation threshold following melatonin inhibition. Such sex-linked neuroendocrine plasticity could be valuable for managing fertility timing in equids, particularly under subtropical conditions with minimal photoperiod variation. In practice, initiating evening photostimulation 6–8 weeks before breeding could accelerate testicular reactivation and enhance semen quality, as observed in other species. [22,24]. A combined regimen using blue light (for pineal inhibition) and red light (for gonadal activation) may provide optimal results without overexposure, reflecting physiological synergy as demonstrated here (Table 1); (Figure 4).

While this study robustly demonstrated endocrine activation, direct assessment of central neuroendocrine parameters (e.g., GnRH pulse frequency or hypothalamic receptor expression) was not feasible. Hence, mechanistic inferences are based on peripheral hormonal and statistical evidence. Nevertheless, the strong alignment of hormonal correlations and PCA components (Table 2) confirms the reliability of the observed light-induced modulation of melatonin suppression and HPG activation.

Although the sample size was moderate, effect sizes for key traits were substantial and biologically meaningful. Future studies should assess sperm quality, perform testicular ultrasonography, and longitudinally assess fertility outcomes to determine whether these endocrine changes translate into improved reproductive performance. Further refinement of light intensity, duration, and wavelength parameters will be necessary to optimize species-specific photo-stimulation protocols.

The current findings validate photo-stimulation in male donkeys via The Neuroendocrine pathway: Ocular light suppresses melatonin, relieving GnRH inhibition and triggering pituitary release of LH and FSH. Photo-stimulation reproduces the endocrine milieu of the natural breeding season. The behavioral and endocrine synchronization achieved here mirrors the seasonal reactivation observed in other long-day species [22,29,36]. This research demonstrates the feasibility of regulating seasonal reproduction in donkeys through targeted light manipulation that combines photoperiodic entrainment and photo-biomodulation. These findings are the first step towards the design of improved lighting systems, the management of energy, and the further integration of night biology with livestock production for the benefit of producers and the environment.

## 5. Conclusions

The results validate the statement that spectral Equilume^®^ masks can inhibit melatonin and activate the pituitary-gonadal axis, thereby enhancing the metabolic function of male Dezhou donkeys during the non-breeding season. Specifically, red light (625–650 nm) has the highest efficiency for activating the endocrine system, whereas blue light (468 nm) yields the best results for pineal suppression. Photoperiod is not the only factor affecting seasonal breeding in donkeys. Circadian rhythms, the circadian clock, and geographical location together affect the neuroendocrinology of seasonal reproduction in donkeys, in which melatonin and photoperiod are the main players modulating this canvas. Further research should include body weight and deeper molecular mechanisms to better explore the effect of various monochromatic lights on reproductive efficiency during non-seasonal breeding in donkeys.

## Figures and Tables

**Table 1:** Descriptive summary of endocrine responses to different light spectra in male donkeys (Mean ± SE).

Hormone	A	B	C	D	*p*-Value	Direction of Change vs. Control
FSH (mIU/mL)	↑ 1.65 ± 0.10	1.29 ± 0.07	1.36 ± 0.09	1.12 ± 0.08	**0.032**	↑ under red, moderate ↑ white
LH (mIU/mL)	↑ 3.20 ± 0.22	2.75 ± 0.18	2.88 ± 0.19	2.45 ± 0.15	**0.045**	↑ under red > white > blue
Testosterone (ng/mL)	↑ 5.40 ± 0.42	3.90 ± 0.35	4.20 ± 0.38	3.50 ± 0.30	**0.021**	↑ red > white
Melatonin (pg/mL)	60.3 ± 3.8	55.2 ± 3.2	58.8 ± 4.1	82.4 ± 4.5	**0.018**	↓ under all artificial lights
AMH (ng/mL)	↑ 1.10 ± 0.06	0.97 ± 0.04	1.15 ± 0.05	0.92 ± 0.05	**0.041**	↑ white and red
Inhibin B (pg/mL)	↑ 90.8 ± 5.6	80.4 ± 4.8	84.6 ± 5.3	72.1 ± 5.0	**0.036**	↑ under all treatments
Activin A (pg/mL)	↑ 74.6 ± 4.9	70.3 ± 5.1	68.9 ± 4.6	62.5 ± 4.3	**0.049**	↑ red > blue > white
P4 (Pmol/L)	511.89 ± 35.12	532.62 ± 45.53	524/85 ± 39.81	574.81 ± 23.39	0.25	Moderate ↑ blue ↑ under red and white light

Note: Values represent least-square means ± standard error of the mean. Bold values indicate statistically significant (*p* < 0.05) difference from control. Directional arrows indicate trends of hormonal up- or down-regulation.

**Table 2:** Principal Component Loadings and Variance Explained by Endocrine Variables under Different Light Treatments.

Principal Component	Eigenvalue	% Variance Explained	Most Substantial Positive Loadings (r > 0.70)	Most Substantial Negative Loadings (r < −0.70)	Major Biological Interpretation
PC1	3.84	47.9%	LH, FSH, Testosterone, Activin A, Inhibin B	Melatonin	Reproductive axis activation vs. circadian inhibition
PC2	1.92	23.7%	AMH, Inhibin B		Sertoli function and metabolic coupling
PC3	1.02	12.8%	Melatonin,	—	Light-driven oxidative–endocrine modulation
PC4	0.65	8.1%	—	—	Residual variance

Note: Principal component analysis (PCA) was conducted on z-scored hormone data (*n* = 12 males). Components with eigenvalues > 1 were retained. Loadings >|0.70| are considered biologically meaningful.

## Data Availability

The original contributions presented in this study are included in the article. Further inquiries can be directed to the corresponding author.
